# Diagnosis and Monitoring of Achalasia Utilizing Point-of-Care Ultrasound (POCUS): A Case Report

**DOI:** 10.7759/cureus.77308

**Published:** 2025-01-12

**Authors:** Yupar Linn, Phyu Phyu Han, Kian Chai Lim, Vui H Chong

**Affiliations:** 1 Department of Medicine, Pengiran Muda Mahkota Pengiran Muda Haji Al-Muhtadee Billah (PMMPMHAMB) Hospital, Tutong, BRN; 2 Department of Radiology, Pengiran Muda Mahkota Pengiran Muda Haji Al-Muhtadee Billah (PMMPMHAMB) Hospital, Tutong, BRN; 3 Department of Radiology, Raja Isteri Pengiran Anak Saleha (RIPAS) Hospital, Bandar Seri Begawan, BRN; 4 Department of Medicine, Raja Isteri Pengiran Anak Saleha (RIPAS) Hospital, Bandar Seri Begawan, BRN

**Keywords:** achalasia cardia, esophageal achalasia, monitoring, point-of-care ultrasound, ultrasound diagnosis, ultrasound (u/s)

## Abstract

Achalasia is a rare disorder affecting the esophagus, in which the lower esophageal sphincter (LES) is spastic and fails to relax, causing food retention. This leads to esophageal dilatation over time. The condition often presents late and is frequently not suspected, resulting in delayed diagnosis. Traditionally, it is diagnosed through manometry, which shows failure of relaxation of the LES, along with ineffective or absent peristalsis. Barium swallow imaging reveals the pathognomonic "rat’s tail" sign and a dilated esophagus. We report an interesting case of achalasia that was initially suspected on chest radiography, diagnosed using point-of-care ultrasound (POCUS), and later confirmed with a computed tomography scan. The patient improved after two sessions of balloon dilation, and monitoring with POCUS showed improvement. This case highlights the potential role of POCUS in both the diagnosis of achalasia and in monitoring after treatment.

## Introduction

Achalasia is a relatively rare motility disorder of the esophagus, with an annual incidence of one per 100,000 individuals and a prevalence of 10 per 100,000 [1]. It is caused by the degeneration of the myenteric nerve plexus in the lower esophagus, leading to spasms of the lower esophageal sphincter (LES). This results in the failure of LES relaxation and a loss or reduction of esophageal peristalsis during swallowing. These abnormalities create a functional obstruction at the gastroesophageal junction (GEJ), which leads to esophageal dilation. The underlying etiology is multifactorial, potentially involving infections (e.g., herpes simplex virus), autoimmune disorders, and genetic factors [2]. The condition affects both men and women equally. An association with malignancy leading to infiltration of the LES is known as pseudoachalasia [3]. Achalasia carries several risks of complications, including megaesophagus, recurrent aspiration pneumonia, and esophageal cancer.

Diagnosis is typically achieved through imaging techniques such as barium swallow studies and endoscopy, but the definitive diagnosis is made through manometry, especially in early cases. Pseudoachalasia needs to be excluded. Treatment options include endoscopic balloon dilation, botulinum toxin injections, myotomy (either surgical Heller’s myotomy or peroral endoscopic myotomy), and esophagectomy [1,2,4]. After treatment, patients are monitored for symptom improvement and, definitively, through barium swallow studies.

We report an interesting case of achalasia that was suspected after a plain chest radiograph, diagnosed, and monitored after treatment using point-of-care ultrasound (POCUS), highlighting the important role of ultrasound or POCUS in managing achalasia.

## Case presentation

A 42-year-old woman with a history of hypertension and dyslipidemia was admitted due to two weeks of vomiting after eating. On the day of admission, she vomited a small amount of blood, which was preceded by nausea, chest discomfort, and mild epigastric pain. Further questioning revealed that she had experienced progressive difficulty swallowing over the past three months, initially with solid foods and later including liquids. To alleviate her symptoms, she adjusted her diet from normal to soft foods and then to liquids. She denied any odynophagia, coughing, especially after meals, or any lower gastrointestinal symptoms. Additionally, she reported some weight loss during this period. There was no history of smoking or alcohol use.

**Figure 1:**
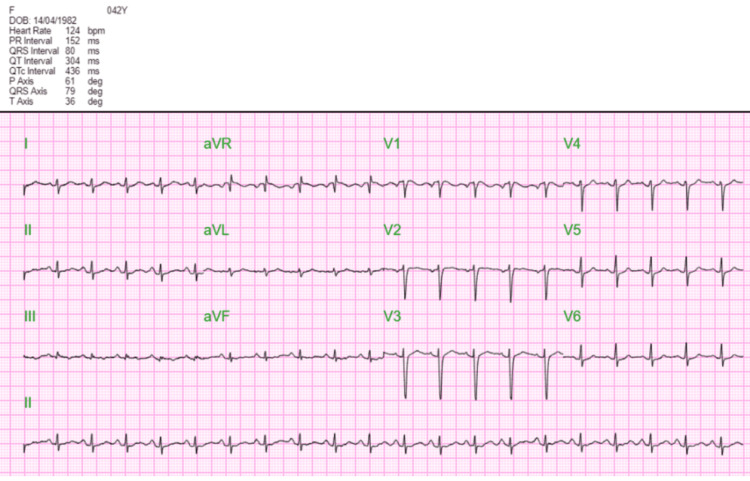
Electrocardiogram done on admission, showing only sinus tachycardia aVL: augmented vector left; aVR: augmented vector right; aVF: augmented vector foot

On physical examination, she had a small body frame and a tachycardia of 105 beats per minute. Chest, cardiac, and abdominal examinations were normal. Laboratory investigations revealed leukocytosis (12.4 x 10^3^/µL, normal range: 4.2-12.6), a normal serum hemoglobin (14 g/dL, normal range: 11.5-15.9), and thrombocytosis (528 x 10^3^/µL, normal range: 174-430). Electrolytes and liver enzymes were within normal limits, except for a mildly elevated creatinine level (102 µmol/L, normal range: 44.2-97.2). Thyroid function tests were normal. An electrocardiogram done in the emergency department earlier showed sinus tachycardia without ischemic changes (Figure 1). Echocardiography that was done later was normal: ejection fraction was 77.5%, and no regional wall motion abnormality was noted.

The patient was managed for a Mallory-Weiss tear against the backdrop of probable chronic gastroesophageal reflux disorder. She was kept nil per os, started on intravenous fluids, and given intravenous omeprazole and metoclopramide. An upper gastrointestinal endoscopy was planned.

**Figure 2:**
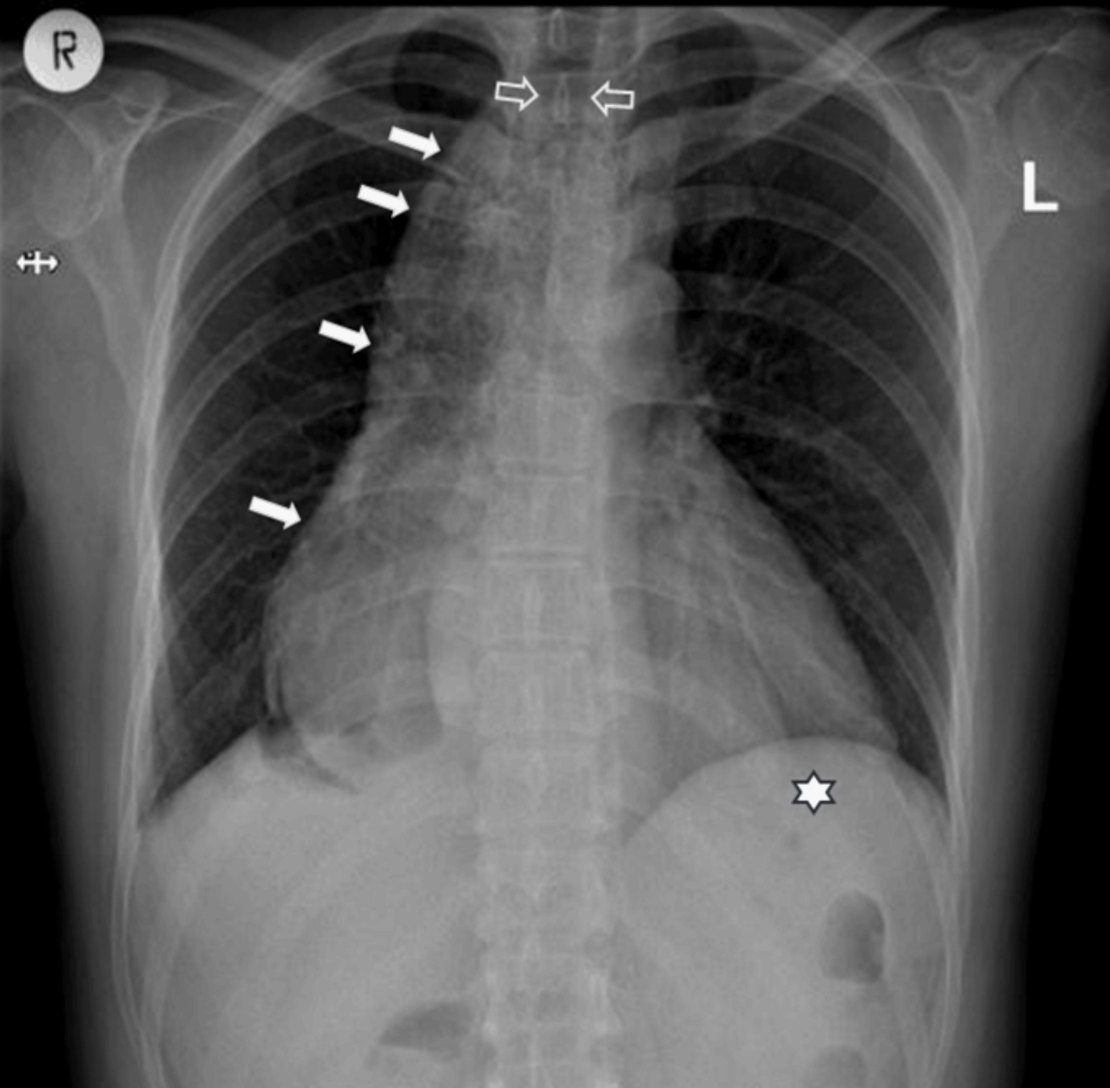
Plain chest radiograph showing a convex widening of the right mediastinal border (solid arrows), with air-fluid level (open arrows) suggestive of a grossly dilated esophagus, and absent gastric bubble (*)

A chest radiograph was done, and this showed a widened mediastinum from a dilated esophagus with an absent gastric bubble, suspicious of achalasia (Figure 2).

**Figure 3:**
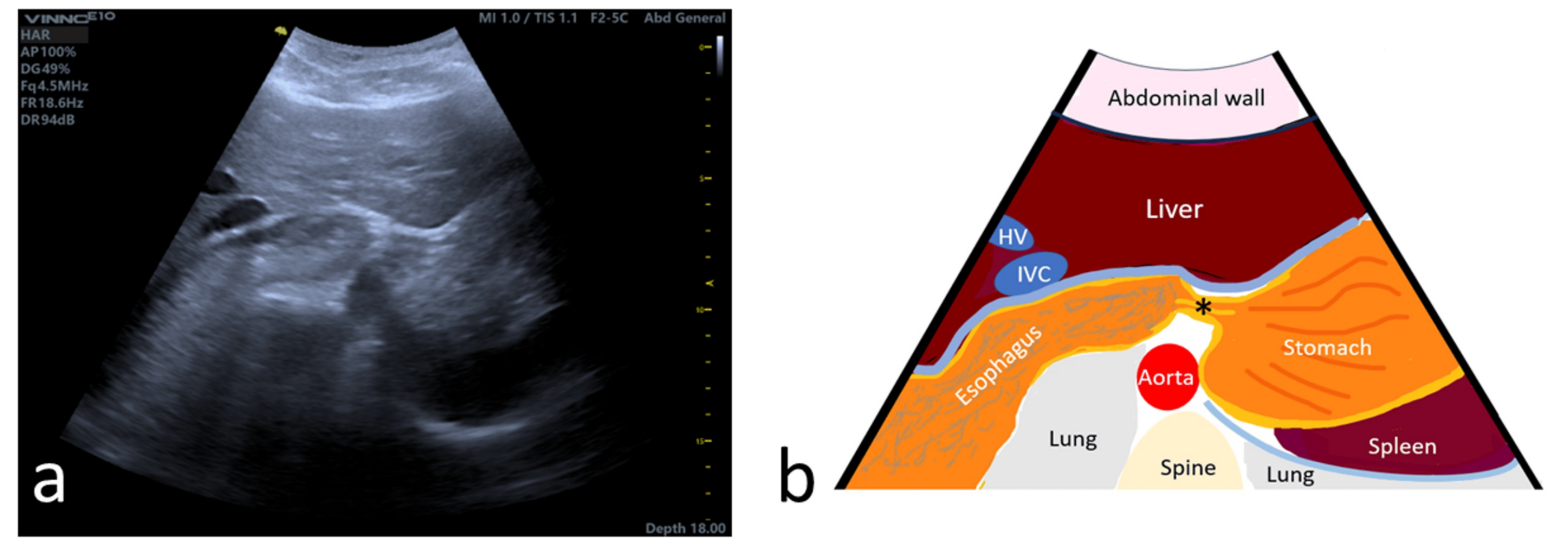
POCUS at the epigastrium level using the liver as acoustic window showing (a) dilated distal esophagus with fluid content located posterior to the liver. (b) Corresponding drawing illustration of the POCUS image POCUS: point-of-care ultrasound; IVC: inferior vena cava; HV: hepatic vein *Gastroesophageal junction Image credit: Figure 3b is an original image created by the author Vui H. Chong

A bedside POCUS was performed with the probe placed at the epigastrium, using the liver as a window. This revealed a dilated structure located behind the posterior border of the liver (Figure 3), with fluid contents sloshing around with respirations and patient movements, consistent with a dilated esophagus. The distal end tapered to a cigar-shaped form, with the GEJ clearly visualized in some views. The rest of the upper abdominal structures (pancreas, liver, stomach, gallbladder, spleen, and kidneys) appeared normal. A subcostal examination of the heart revealed normal cardiac function with no other abnormalities.

**Figure 4:**
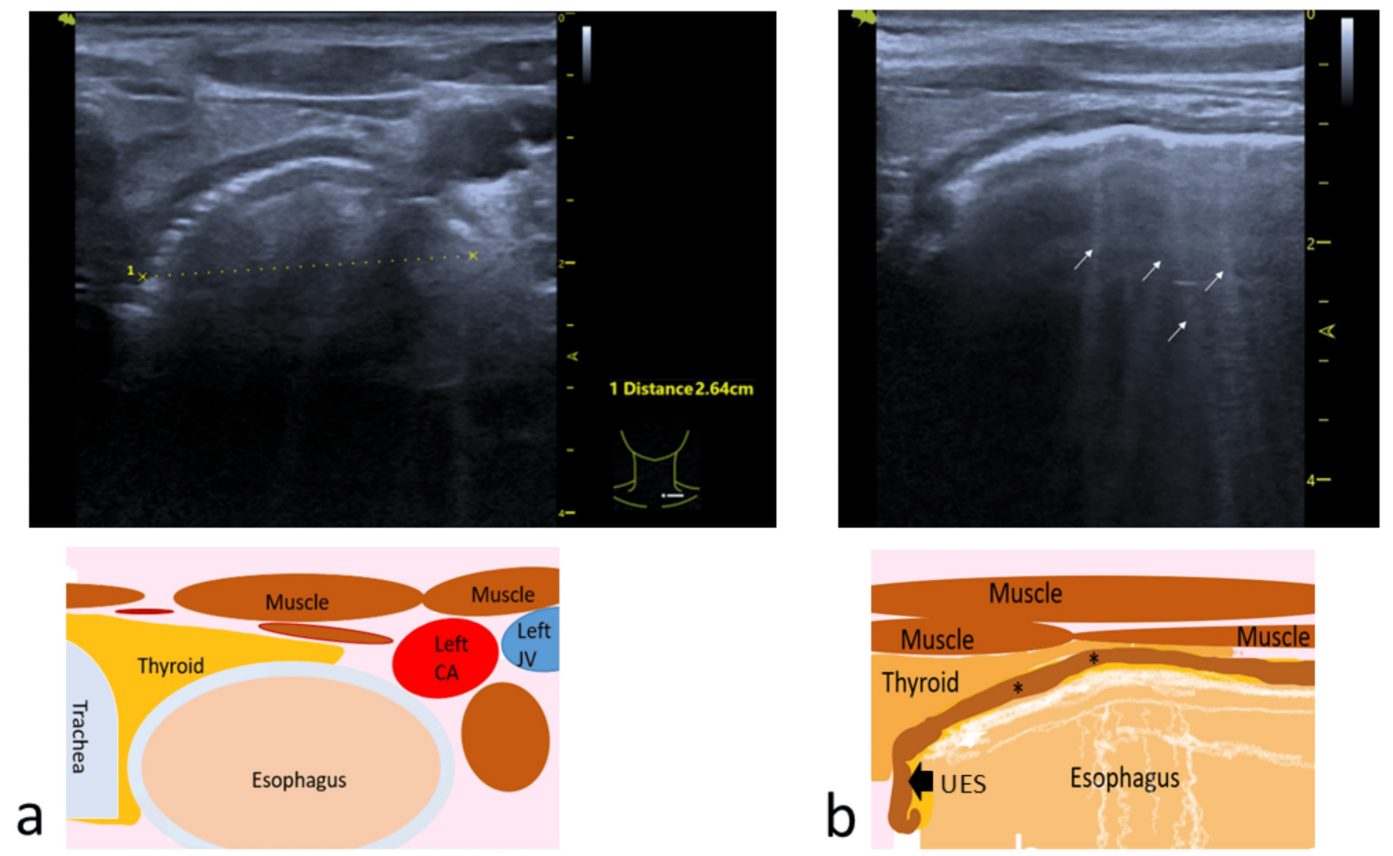
POCUS at the cervical esophagus. (a) Transverse view showing a dilated cervical esophagus measuring 26.4 mm with reverberation artifacts due to air inside the esophagus with corresponding drawing illustration showing the structures. (b) Sagittal view of the scan showing a dilated esophagus up to the UES and the corresponding drawing illustration showing the structures. Reverberation artifacts due to air inside the esophagus highlighted by small white arrows and thickened esophageal wall (*) POCUS: point-of-care ultrasound; UES: upper esophageal sphincter Image credit: The corresponding drawing illustrations are original images created by the author Vui H. Chong

POCUS examination at the neck level showed a dilated cervical esophagus up to the level of the upper esophageal sphincter (UES) in both transverse and sagittal views (Figure 4). The dilated esophagus measured 26.4 mm.

**Figure 5:**
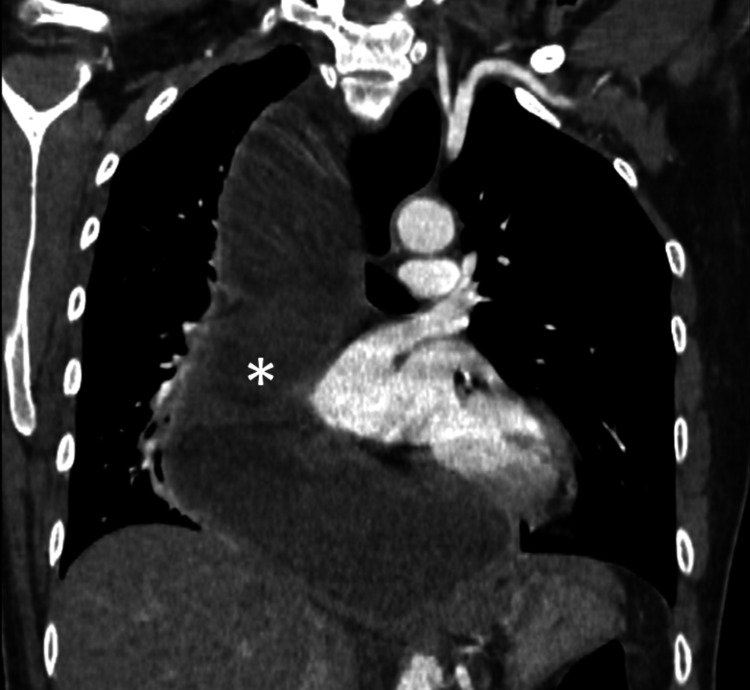
Coronal CT image showing a grossly dilated fluid-filled esophagus (*) extending up to the neck CT: computed tomography

Computed tomography of the chest confirmed the findings of achalasia with megaesophagus, showing an air-fluid level on the axial images and a mega- or sigmoid-shaped esophagus on the reconstructed coronal image (Figure 5).

**Figure 6:**
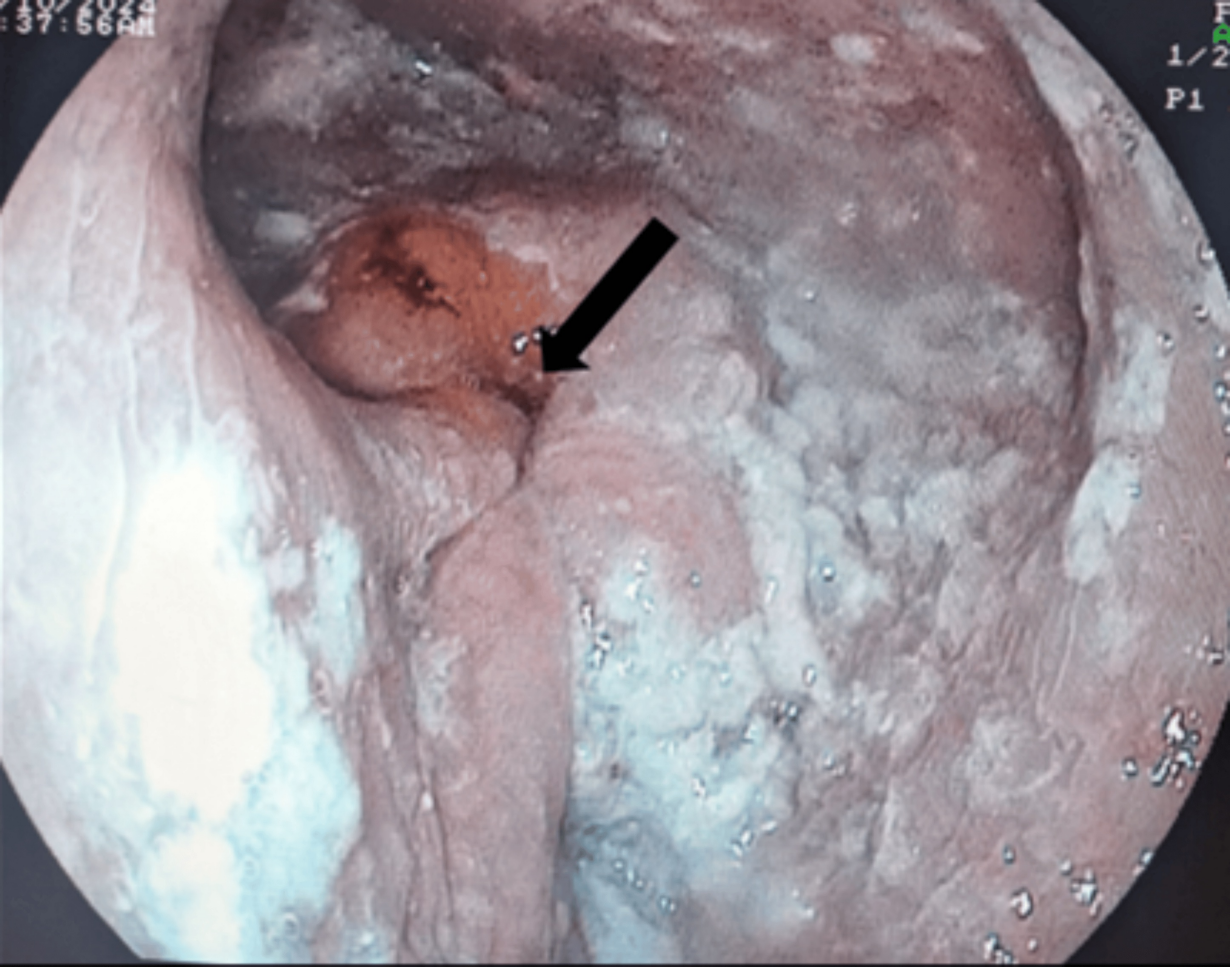
Upper gastrointestinal endoscopy showing a dilated esophagus with food residue and lower esophageal sphincter spasm, prior to balloon dilation. Gastroesophageal opening is indicated by an arrow

The patient was kept nil by mouth for 24 hours to decompress the esophagus and continued on the same treatment regimen. Additionally, nifedipine was added to the treatment. The patient then underwent upper gastrointestinal endoscopy and esophageal balloon dilation. A copious amount of fluid and food debris were cleared before dilation (Figure 6). The GEJ was dilated with a through-the-scope 20-mm controlled radial expansion balloon dilator. This was insufflated with air. A further attempt to dilate with an over-wire 30-mm balloon was not possible due to the grossly dilated esophagus, making passage of the dilator through the GEJ difficult. Gastric examination revealed hemorrhagic gastritis, and testing for *Helicobacter pylori* was positive.

After the dilation, the patient tolerated food better and was started on eradication therapy. On follow-up two weeks after balloon dilation, her symptoms had improved, but POCUS showed no change in the distal and diameter of the cervical esophagus. The patient then underwent further balloon dilation with a 30-mm balloon dilator in the left lateral position. The balloon was insufflated with diluted contrast, and its position was assessed using a combination of fluoroscopy and POCUS. For the POCUS scan, the probe was placed in the epigastrium and adjusted to visualize both the balloon and the GEJ.

After dilation, the patient's symptoms markedly improved, with near resolution of her symptoms. POCUS monitoring showed a reduction in the cervical esophageal diameter compared to the measurement at diagnosis (Table 1). The patient remained well and symptom-free at follow-up.

**Table 1:** POCUS assessment of the distal and cervical esophagus at diagnosis and post balloon dilations POCUS: point-of-care ultrasound

Variables	Diagnosis	Two weeks after the first balloon dilation	Two weeks after the second balloon dilation	Six weeks after the second balloon dilation
Cervical esophagus: measurement in transverse scan	26.4 mm	No improvement	13.7 mm	10.8 mm
Distal esophagus	Grossly dilated	No improvement	Regression	Further regression

## Discussion

**Figure 7:**
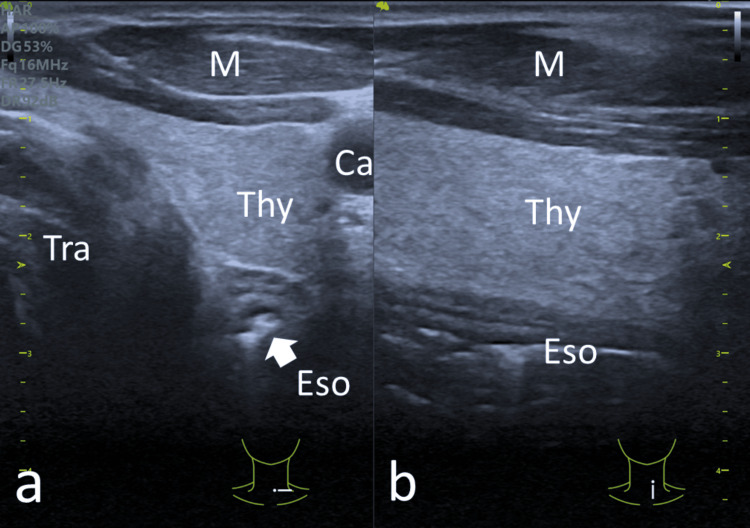
POCUS of a normal cervical esophagus located posterior to the left lobe of the thyroid gland in the (a) transverse view appearing as a ring structure (white arrow) and (b) sagittal view appearing as a layered tubular structure with reverberation artifacts from content inside the esophagus POCUS: point-of-care ultrasound; Eso: esophagus; Tra: trachea; Thy: thyroid; Ca: carotid artery; M: muscles

We report an interesting case of achalasia, which was diagnosed using POCUS. Additionally, POCUS played a crucial role during balloon dilation and in monitoring the treatment response. The POCUS scans were performed at both the epigastric and cervical levels. At the time of diagnosis, they revealed a dilated esophagus extending to the UES. In our case, achalasia was easily diagnosed due to the severity of the disease. This case demonstrates that achalasia of the esophagus can be detected and diagnosed with POCUS; however, the esophagus must be sufficiently dilated for this to be appreciated. In a normal esophagus, the distal esophagus is visible for a short distance above the GEJ, and the cervical esophagus, located at the posterior surface of the thyroid lateral to the trachea, appears as a ringed structure in the transverse scan and as a layered structure in the sagittal scan (Figure 7).

Traditionally, achalasia is diagnosed with manometry and barium swallow [1,2,4], with high-resolution manometry being the gold standard. This is particularly true for the early stage of the disease before the esophagus becomes dilated. Barium swallow typically shows (a) a dilated esophagus, with the degree of dilation depending on the duration of the disease, ranging from mild dilation to a tortuous, dilated sigmoid esophagus resembling a sigmoid colon, (b) a rat-tail sign at the GEJ due to spasm of the LES, and (c) tertiary contractions in some cases [2,4].

To date, there are only a few reports on ultrasound or POCUS in the context of achalasia [5-10]. Several studies have reported ultrasound features of achalasia and compared them to other diseases of the esophagus and normal subjects [5,6]. One study reported that in achalasia (4.8 ± 0.9 mm), the GEJ is thicker than in normal subjects (2.0 ± 0.5 mm) but significantly less compared to patients with pseudoachalasia or carcinoma (19.0 ± 1.1 mm) [5]. Another study reported thicknesses of 3.3 ± 1.2, 5.1 ± 2.3, and 19.5 ± 7.8 mm for normal volunteers, achalasia, and malignancies, respectively [6]. In malignancies, the thickness of the GEJ is often not consistent and has irregular borders [5]. Interestingly, none of these reports have addressed the proximal esophagus. The cervical esophagus can be easily assessed using a linear probe, a normal esophagus will be empty, and its transverse diameter measures a mean of 11.1 ± 1.6 mm (range: 7.1-13.9) [10]. For severe disease, the proximal esophagus is also grossly dilated. In our case, the cervical esophagus measured 26.4 mm at diagnosis, and the dilation could be seen extending to the UES.

Interestingly, the middle portion of the esophagus can also be visualized on sonographic imaging using the heart as a window [11]. In this interesting case, a 65-year-old man who presented with symptoms of worsening dysphagia, regurgitation, and weight loss had an echocardiogram that showed an extracardiac mass that was located posteriorly, compressing the left atrium [11]. Similar to our case, a chest radiograph showed a widened mediastinum, and a barium swallow diagnosed achalasia. This case highlighted that the middle portion of the esophagus can also be visualized with ultrasound.

Apart from detection and diagnosis, POCUS can also be used to monitor treatment with balloon dilation and treatment response. When POCUS is used, the patient should be positioned in the left lateral position for easy access to the epigastrium, and the balloon should be insufflated with a liquid, such as liquid contrast, to provide a good acoustic window. Importantly, POCUS can also be used to monitor treatment response. This reduces unnecessary radiation exposure from barium swallow, which is typically used for monitoring treatment response. In our case, follow-up with POCUS showed interval reductions in the dilation of the esophagus.

POCUS is increasingly being incorporated into clinical practice. While its use is widely accepted in certain specialties, such as emergency and trauma medicine, anesthesia, critical care, and pediatric surgery, its adoption remains limited in other fields [12-15]. POCUS is a valuable, portable tool that provides instant information, which can significantly influence management decisions. However, there are limitations that must be acknowledged. Access to probes or ultrasound machines may be constrained due to the added cost, and POCUS also requires training in both image acquisition and interpretation. As more clinicians begin to adopt this tool, it is crucial that they gain sufficient experience, as misinterpretations can lead to undesirable outcomes with serious consequences. Fortunately, many resources and training courses are now available, offering curricula designed to ensure practitioners acquire the necessary knowledge.

## Conclusions

We report an interesting case of severe achalasia in which POCUS played a major role in its management, from diagnosis and monitoring the position of the balloon during dilation to tracking treatment response. Due to the severity of the disease in our case, esophageal dilation was easily observed at both the distal and cervical levels. In milder cases, detection and diagnosis may be difficult or not possible with POCUS. Importantly, the use of POCUS for treatment response avoids the radiation exposure associated with radiological imaging, such as barium swallow, which is typically used to assess treatment response. This case highlights the importance of POCUS in clinical practice, not only for its established uses but also for its expanding applications. The advantages of POCUS include its portability, ability to provide instant information, and, importantly, its lack of radiation exposure.
